# *Balancing the cost of leaving with the cost of living:* drivers of long-term retention of health workers: an explorative study in three rural districts in Eastern Uganda

**DOI:** 10.1080/16549716.2017.1345494

**Published:** 2017-08-25

**Authors:** Suzanne Namusoke Kiwanuka, Martha Akulume, Moses Tetui, Rornald Muhumuza Kananura, John Bua, Elizabeth Ekirapa-Kiracho

**Affiliations:** ^a^ Makerere University School of Public Health (MakSPH), Department of Health Policy Planning and Management, Makerere University, Kampala, Uganda; ^b^ Epidemiology and Global Health Unit, Department of Public Health and Clinical Medicine, Umeå University, Umeå, Sweden

**Keywords:** MANIFEST: Maternal and Neonatal Implementation for Equitable Systems Study, Human resource management, retention, implementation science, Uganda

## Abstract

**Background**: Health worker retention in rural and underserved areas remains a persisting problem in many low and middle income countries, and this directly affects the quality of health services offered.

**Objective**: This paper explores the drivers of long-term retention and describes health worker coping mechanisms in rural Uganda.

**Methods**: A descriptive qualitative study explored the factors that motivated health workers to stay, in three rural districts of Uganda: Kamuli, Pallisa, and Kibuku. In-depth interviews conducted among health workers who have been retained for at least 10 years explored factors motivating the health workers to stay within the district, opportunities, and the benefits of staying.

**Results**: Twenty-one health workers participated. Ten of them male and 11 female with the age range of 33–51 years. The mean duration of stay among the participants was 13, 15, and 26 years for Kamuli, Kibuku, and Pallisa respectively. Long-term retention was related to personal factors, such as having family ties, community ties, and opportunities to invest. The decentralization policy and pension benefits also kept workers in place. Opportunities for promotion or leadership motivated long stay only if they came with financial benefits. Workload reportedly increased over the years, but staffing and emoluments had not increased. Multiple job, family support, and community support helped health workers cope with the costs of living, and holding a secure pensionable government job was valued more highly than seeking uncertain job opportunities elsewhere.

**Conclusion**: The interplay between the costs of leaving and the benefit of staying is demonstrated. Family proximity, community ties, job security, and pension enhance staying, while higher costs of living and an unpredictable employment market make leaving risky. Health workers should be able to access investment opportunities in order to cope with inadequate remuneration. Promotions and leadership opportunities only motivate if accompanied by financial benefits.

## Background

Effective delivery of health services requires the availability of adequate numbers and skill mix of health workers. The challenge of attaining this ideal is most critically observed in rural settings [1]. The attraction and retention of health-care professionals in rural and underserved areas is essential in ensuring that these areas acquire equitable care [2]. However, the challenge of retaining health workers in rural and underserved areas persists [3]. For several reasons such as availability of amenities, better transport, accommodation, and utilities (among other things), most health workers prefer to work in urban areas [4]. Hence rural posting often does not lead to the expected retention of workers [5]. In-country migration of health workers from rural to urban areas, leaves rural areas grappling with both low staffing levels and under qualified staff [6].

**Figure 1.**
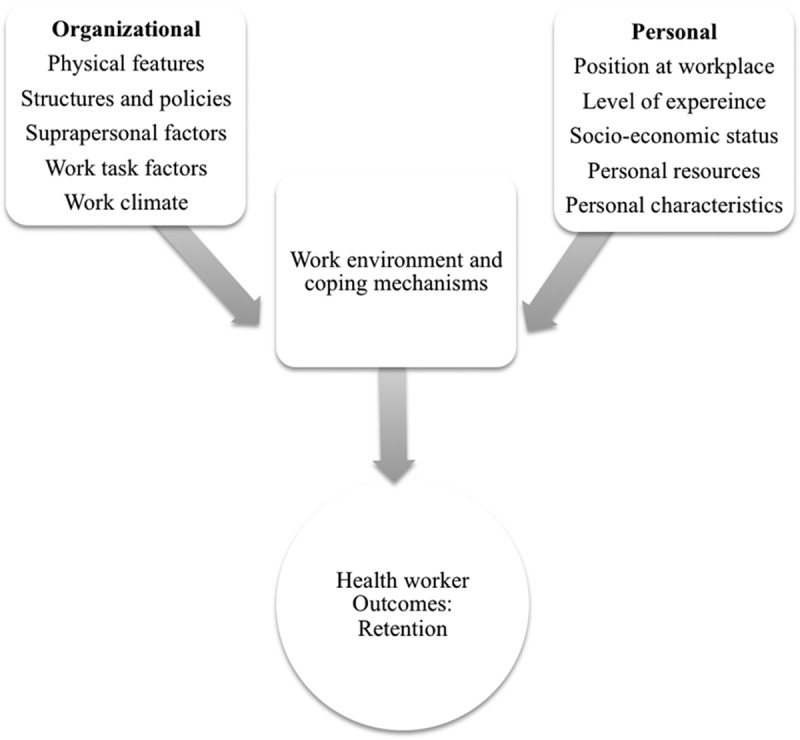
Health worker retention framework adapted from Schaefer and Moos [22].

Uganda’s health worker mal-distribution has persisted, despite attempts by government to mitigate them. In hard-to-reach areas these challenges are particularly magnified due to poor motivation and high rates of attrition [7]. Uganda has a population of approximately 37 million people as of 2014, 75% of who live in rural areas. The highly trained human resource, including medical doctors, degree and specialized nurses/midwives, pharmacists, dentists, as well as diagnostic personnel, are unequally distributed, serving only a fraction of the population [8]. Approximately 70% of Uganda’s health workers are found in urban areas where 27% of the population lives. The central region, which only hosts 27% of the population [9] has the biggest proportion of skilled health professionals, including 64% of all nurses and midwifery professional cadres (degree holders and specialist registered nurses), 71% of all medical doctors, 76% of all dentists, and 81% of all pharmacists.

These health worker challenges have resulted in a health workforce faced with increased workloads amidst other resource constraints and consequently suffering low motivation, poor performance, and deterioration in quality of care and health sector performance. At the moment, the number of women who die as a result of pregnancy or child-bearing related causes in the country stands at 438 per 100 000 live births. These statistics are largely underpinned by rural-urban disparities. Most maternal deaths occur among mothers in rural areas [10], and the latest reports show that about 36% of pregnant women in rural areas deliver under a skilled attendant, the rest resorting to traditional birth attendants [11].

### Health worker retention in Uganda

Health worker retention is defined as the health workers’ decision to stay in practice [12]. The low numbers of health workers in rural areas reflect more of a retention problem rather than a recruitment one [13]. According to the Ministry of Health [14], only 7211 of the 8353 health workers recruited in 2013 reported to work. In the same year, some facilities lost 25% of their staff. For three years, the MANIFEST project provided training, support supervision, and mentorship with the aim of motivating health workers towards quality service delivery for maternal and child health [15]. In some instances, health workers were lost following training and hence the capacity built was compromised by these losses.

This loss directly affects the quality of health services offered and ultimately has consequences for service uptake. The government of Uganda has made several attempts to address the issue of health worker retention and motivation [16]. These have been embedded in both national policies as well as locally designed strategies. These strategies have been described in detail, and include civil service reforms and decentralization (1993), pay reforms and salary increments (1995), the centralization of payroll for health workers (2001/2002), the consolidation of lunch allowances (2003), the Uganda Human Resources for Health Policy, the Uganda Human Resources for Health Strategic Plan (2005–2020), the motivation and retention of human resources for health (2008), the hard to reach allowances policy (2010), and the salary increment for doctors at Health Centre IVs (HCIVs) in Uganda, 2012 [17]. Local governments have also instituted incentives to boost motivation and retention, which have included regular monthly and quarterly support supervision. The rewards and sanctions scheme, accommodation for health workers, rotational transfers, training opportunities, and tea breaks were implemented to reward and sanction health workers. However, this plethora of government and locally engineered strategies for motivation appear to work better for some health workers compared to others as shown in other settings [18–21].

Amidst this mal-distribution and high turnover, some health workers are retained in rural underserved areas for long periods of time. This particular brand of health worker offers lessons for future policy and practice in designing retention incentives in underserved areas. This paper explores the drivers of long-term retention and describes health worker coping mechanisms in rural areas and underserved areas.

The study adapted a conceptual model of Organizational and Personal Factors and Outcomes, by Schaefer and Moos [22] to explore the drivers of long term retention among health workers in rural Uganda  (see Figure 1). According to this model, the organizational system comprised of: physical features, structures, policies, work climate, supra-personal factors, and coping influence retention. The personal factors nclude; the characteristics of individuals, such as their position at work and level of experience, socio-demographic background, personal resources, and expectations and preferences about the workplace. Furthermore, work stressors combined with organizational and personal system factors can also influence coping responses and employee outcomes. As a result of these influences, employees work morale and performance and retention are affected [19,21–24]. This study aimed to explore the organizational, work environment, and personal factors that may influence long-term stay and to describe health worker coping mechanisms in rural settings. The strategies identified could be incorporated into interventions that are aimed at bringing about long-term changes in the health workforce.

## Methods

### Study area

The study was conducted in three rural districts Kamuli, Kibuku, and Pallisa. Kamuli is located 96 km east of Kampala, the capital of Uganda, while Kibuku and Pallisa are 200 km north-east of Kampala. Kamuli has a population size of about 486 319, Kibuku 202 033, and Pallisa about 386 890 people [25]. The three districts have 104 health facilities, 33 in Pallisa, 17 in Kibuku, and 54 in Kamuli [25].

According to the Ministry of Health [26], less than three out of five of approved health-worker posts were filled in the districts. Pallisa District had the largest proportion of filled approved posts (60%) and Kamuli District had the least proportion (49%) of approved filled posts. Table 1 provides a description of the study area.

### Study design

This was a qualitative study that explored the lived experiences of health workers that motivated them to stay and work in rural facilities. The data was collected through in-depth interviews with 21 out of 34 health workers retained for at least 10 years in the study districts, as of July 2015.

### Sampling and data collection

The District health officers generated a list of health workers retained for more than 10 years. Four research assistants used telephone contact to secure interviews with 25 health workers, however only 21 telephone interviews were conducted during the month of July 2015 due to the busy schedules of some of the respondents. A structured open-ended interview guide in English language was used to collect data. The interview guide included questions on working environment (staff housing, availability of schools), organizational leadership, recognition and incentives, and other personal factors (such as place of birth, family residence). The interviews were conducted in English language and each interview took 30–45 minutes. The interview guides were structured in such a way that interviewers could write the responses for each question under its designated space on the interview guide during the interview.

### Participants’ characteristics

In-depth interviews were conducted among 21 health workers, 10 were male and 11 were female. The health workers’ age ranged from 33 to 55 years. Nine of them were senior nursing officers, two were clinical officers, six were senior nursing officers, and four were midwives. Regarding the duration of stay, health workers from Pallisa district had longer duration in service, with a mean of 26 years (Table 2).

**Table 1.** Description of study area.

District	Kamuli	Pallisa	Kibuku
Area in sq. km	1557	1461	490
Distance from Kampala, capital city	142	195	182
Number of sub-counties	5	12	10
Antenatal care attendance (4 visits) **	66%	64%	57%
Deliveries**	58%	74%	74%
Postnatal care attendance**	70%	63%	64%

Source: *Uganda Bureau Of Statistics (2015), **MANIFEST evaluation (2015)

**Table 2.** Participants’ characteristics.

Characteristics	Kamuli	Pallisa	Kibuku
Male/Female	3/5	4/3	3/3
Age range (years)	34–54	45–51	33–47
Mean duration in service (years)	13.3	26.4	15.2

### Data analysis

The deductive context analysis approach [27] was used for analysis, with the following stages: coding the transcribed data from the interview; identifying categories from the interview scripts; grouping the categories into major predetermined themes (personal, organizational, work stress, and coping strategies); deriving meaning under each of these themes to explain the findings. Table 3 provides examples of analyses processes.

**Table 3.** Examples of analyses processes.

Text	Codes	Categories	Theme
*ʽDecentralization and internal transfers have proved to be a challenge. So you stay here with your qualifications, but they don’t promote you because they have no funds-and it’s hard to move to another district because the payroll gets derailed and you might not receive salary in a long timeʼ:* Female health worker in Kamuli district	Decentralization Internal transfers no funds promotion pay-roll salary	Recruitment Policies Non-financial incentives Financial incentives	Organizational factors
*ʽI only wanted to work in a public facility, but because I am in a rural place, I also do agriculture. Being at home among my people and having a clinic I go to during the weekend and off days is very motivatingʼ:* male health worker in Pallisa district.	Agriculture At home My people Clinic Off days	Financial Non-financial incentives Call to serve	Organizational Personal Coping mechanisms
*ʽI had an opportunity to work in the private sector in another district which pays twice what I earned here (1 200 000 compared to 600 000), but I knew that staying here I would have an opportunity for promotion in the long run and when I retire I have benefits and when I die my people would get benefitsʼ:* female health worker in Kibuku district.	Promotion Private sector work Retirement benefits	Recruitment Policies Incentives	Organizational

## Results

This section thematically describes the drivers of long-term stay of health workers based on the framework adapted from Schaefer and Moos [22], clearly highlighting factors which may influence health workers to get embedded within their work environment or deter them from departing. The implications of the findings for policy and practice are later discussed.

### Organizational factors

Generally, organizational factors, which included infrastructure, recruitment policies, and provision of incentives had a dyadic influence on health workers’ willingness to stay in rural posts. On the one hand, decentralization and restrictive recruitment policies prevented health workers from departing while the promise of pension benefits motivated them to stay.

Policies and reforms tended to impact on health workers’ stay. In general, none of the health workers had worked in any other district apart from their current district due to the restrictive nature of the decentralization policy. Health workers attributed their lack of opportunity to move freely to the decentralization policy, which restricts their freedom to move from district to district. However, others found the policy advantageous because it enabled them to work in their home area where they could serve their people while benefiting from other opportunities. Being on the government payroll was highly valued. Some were concerned that departing from a district where one is already on the payroll makes them vulnerable to losing their current position and even their salary.

*ʽ* *For me I am a “born” of here. With decentralization it’s better to work at home. I serve my people and I am happyʼ* (Male health worker, Pallisa district).

*ʽ* *Decentralization and internal transfers have proved to be a challenge. So you stay here with your qualifications, but they don’t promote you because they have no funds; and it’s hard to move to another district because the payroll gets derailed and you might not receive salary in a long timeʼ* (Female health worker, Kamuli district).

Job security and the promise of getting a pension were highly regarded as key influencers of retention for health workers and even deterred them from taking up more lucrative offers.

*ʽ* *I had an opportunity to work in the private sector in another district which pays twice what I earned here (1 200 000 (550 USD) compared to 600 000 (225 USD)), but I knew that staying here I would have an opportunity for promotion in the long run and when I retire I have benefits and when I die my people would get benefitsʼ* (Female health worker, Kibuku district).

The availability of staff accommodation was motivating to some because it reduced their travel expenses, enabled them to stretch their meager pay, and boosted family cohesion. Others lauded the benefits of residing in a rural environment where the cost of living is lower than that of urban settings. One health worker reported that urban accommodation could cost as much as five times more than rural accommodation.

*ʽ* *……. a station with no accommodation and you have to spend money to travel to work daily is not good for your finances and familyʼ* (Male health worker, Kibuku district).

However, having staff accommodation was also a source of stress to others because staying near the facility put them at risk of being required to be on duty 24 hours a day, seven days a week due to staffing shortages. For these health workers, working far away from the facility seemed to offer a reprieve from the escalating workload.

*ʽ* *Sometimes accommodation at a facility can demoralize you. There is no break because even when you are off duty patients come and get you from your homeʼ* (Female health worker, Kamuli district).

Health workers tended to be motivated by leadership responsibilities, but only if they were accompanied by additional payments or increased exposure to district administrative and political leaders. Opportunities that come with financial benefits tended to be those tied to ongoing projects within the district. Financial benefits did not extend to those positions related to exclusively clinical oversight positions for service delivery and this tended to cause dissatisfaction among some.

*ʽ* *Being a district health educator, regional facilitator for capacity building for health, and a facilitator for IMCI all come with extra remuneration, which is really goodʼ* (Male health worker, Kibuku district).

*‘I am now in charge, but this is just extra work with no pay. This in-charge business can really stress. People think you are eating their money yet they are not paying you anything extra…these extra responsibilities of being an in-charge. I would have left it already because it’s really a lot of work for nothing’* (Male health worker, Kamuli district).

Work climate, considered in terms of workload, supervision, availability of supplies, and teamwork, tended not to be highlighted as an influencer of long stay, but rather a consolation for health workers to be able to meet their responsibilities. It should be noted, however that issues of whether the health worker could afford to move were not explored, yet these too could have influenced long stay. Almost each health worker interviewed complained of the increase in workload, particularly without concomitant increase in staffing. Sometimes these challenges were compounded by lack of adequate supplies and equipment, making their work even more burdensome.

*ʽ* *The workload has changed too much. We used to see about 30 patients, now we see about 100 per day. Sometimes I wish I could just retireʼ* (Male health worker, Kibuku district).

*“The workload has increased because of my responsibilities. I have to supervise my juniors, do my official work, but also do project workʼ* (Male health worker, Pallisa district).

Health workers have adjusted to drug stock-out by resorting to prescribing out-of-stock drugs and referring clients to private sector providers. However, the plight of poor mothers who are unable to purchase drugs is still a source of discontent.

*ʽ* *The inadequate supplies are demotivating because you can’t reach your full potential. Imagine a whole health center can lack basic equipment like an artery forceps for dressing wounds. Sometimes you want to run away when clients come and you have nothingʼ* (Male health worker, Kibuku district).

*‘When drugs are not available it affects me…….we would prescribe for mothers to go and buy drugs, yet we know these are poor women. I almost left because I saw no reason of being at the facility’* (Female health worker, Kibuku district).

Most health workers indicated that working conditions have not improved in terms of infrastructure, but they make the best of the prevailing situation since conditions are not any better elsewhere. The health workers who stayed accepted their work environment with an attitude of resignation that they could not change much, but also that working in a difficult environment proved their mettle. Moreover, moving to another district because of infrastructure would not really make much difference:

*ʽ* *The place was terrible and dilapidated. It has now been renovated. It did not demotivate me at all….. The fact that I accepted to be deployed here is enough to show that I can work under hard conditions. I have fought hard for this place to reach this extentʼ* (Male health worker, Pallisa district).

### Training and promotion opportunities

Approximately 25 long term (up to 6 months) training opportunities had been accessed by the respondents during their tenure. Males benefited from 50% of these opportunities, but tended to have benefitted more from longer-term training (mean duration 20 months) than females (mean duration 10 months). Thirteen promotions were recorded, eight among males and five among females. Males also tended to hold more leadership positions than females (six versus three) (see Table 4)

**Table 4.** Training, promotion, and leadership opportunities.

	Males	Females
**Cadre**	Six Senior Nursing Officers	Three Senior Nursing Officers
	Two Clinical Officers	Four Nursing Officers
	Two Nursing Officers	Four Midwives
**Promotion obtained**	8	5
**Leadership opportunities**	8	3
**Training opportunities**	Training opportunities > 6 months = 13	Training opportunities > 6 months = 12
Total duration	200 months	113 months
Average training period	20 months	10 months

The respondents indicated that getting promotions was infrequent, but whenever they occurred they boosted their morale. They also emphasized the added value of being able to hold leadership positions, which placed them in a position to obtain additional revenue while still in their workplace. Moreover holding positions in projects also enabled them to interact with district leadership and thereby influence decisions at this level. Others expressed dissatisfaction with in-charge responsibilities that do not come with financial benefits yet put them to task to ensure that the facility performs to the community expectations.

*ʽ* *In a sense, promotion motivates. When one appreciates your work and says you deserve promotion, it motivates you to stay. You are also psychologically satisfiedʼ* (Male health worker, Kibuku district).

*ʽ* *Of course after being promoted in 2009, I am now very happy and very settled. My friend, we have seen people come and get promoted immediately, but we kept cool and waited for our turn. In government you have to be patientʼ* (Male health worker, Pallisa district).

Mentorship was highlighted as an avenue for building skills, but was infrequently mentioned as a motivation to stay in the rural facility. There were few mentorship opportunities within the districts.

*ʽ* *A well known pediatrician encouraged me to treat children and even recommended me for training. This encouraged me to workʼ* (Male health worker, Kibuku district).

*ʽ* *Mentorship has improved under MANIFEST. We now have people who come and support us to do our work. Even our skills have improved….. not like beforeʼ* (Female health worker, Pallisa district).

### Personal factors

By far the most commonly cited reason for long stay within the rural districts was the intention to be near family, maintain community ties and having opportunities to invest and amass assets. Health workers indicated that being near family in their home districts made low salaries bearable because expenditure is reduced. Furthermore, staying within the home district provided an opportunity to practice agriculture and supplement their income. The social support provided by family was crucial in alleviating work stresses.

*ʽ* *My family is with me. My wife works at a nearby health center. This is my home area.ʼ* (Male health worker, Kibuku district).

*ʽ* *I loved my work, but even the cost of living was high, so I needed to work near home so as to save. I have bought 20 acres of land, paid school fees, and built a house for my familyʼ* (Male health worker, Kibuku district).

*ʽ* *I have built houses, bought pieces of land, but mostly, it is to pay school fees for my children. Being able to support my families is the reason I have stayedʼ* (Male health worker, Pallisa district).

All health workers unanimously indicated a preference for working within the public sector, whether it was urban or rural, mostly because of its permanent job positions:

*ʽ* *In the public sector my work is permanent and pensionableʼ* (Male health worker, Kibuku district).

*ʽ* *I only wanted to work in a public facility, but because I am in a rural place, I also do agriculture. Being at home among my people and having a clinic I go to during the weekend and off days is very motivatingʼ* (Male health worker Pallisa district).

Female providers were however more likely than males to mention the ‘call to serve’ as a major factor affecting their long stay.

*ʽ* *…..opportunities made me feel that I need to stay and serve my people, so it was difficult to leave and goʼ* (Female health worker, Pallisa district).

*ʽ* *When I was looking for a job, I wanted to serve the community. I love my job, the community loves me. There is no reason why I should leave my workʼ* (Female health worker, Kibuku district).

### Coping with work-related stress

Family, teamwork, and the unction to serve were highl ighted as sources of relief from work-related stress. Having family near contributed to stability for the health worker, while good teamwork provided relief from the escalating workload. Some health workers coped by not taking up staff accommodation, thereby separating their work life from their private lives.

*‘Workload has increased….where I work the patients are so many and we work up to late. But I love to serve my clients and my people and it is worth it’* (Female health worker, Pallisa district).

*ʽ* *Having no accommodation at the workplace keeps stress at bay. When housed at the workplace you cannot think for yourself. I leave stress at the workplace and stay awayʼ* (Female health worker, Kamulidistrict).

The community was noted to have both positive and negative influences on the health workers’ long-term stay. Some communities were not appreciative of the burden borne by health workers to provide care or even the shortage of resources, while others were more understanding and appreciative.

*ʽ* *The facility is deep in the village; we serve about 200 patients a day with only one midwife. The workload would not be a problem, but the community attitude. They do not appreciate and this, at times, makes me think of leavingʼ* (Female health worker, Kibuku district).

*ʽ* *The community does not help us much because we only see them when they are sickʼ* (Male health worker, Kibuku district).

*ʽ* *Ever since I joined this place I have had no problem with the community. This good relationship has made me continue working in this areaʼ* (Female health worker, Kibuku district).

### Coping with low or delayed salaries

The findings around coping with inadequate salaries provided a mixed message. Some workers did not mind low salaries as long as payments were regular because the cost of living was low. Some engaged in dual practice while others supplemented their income with project work. Others felt salaries were too low and they would leave if given the opportunity for better pay.

*‘…the standard of living is low and one can save.’* (Female health worker, Kibuku district).

*ʽ* *The salary scale at hospital level is favorable for midwives, so I decided to stay.’* (Female health worker, Pallisa district).

*ʽ* *I can’t say that any thing is keeping me here. I have had no other option. If I got a job of better pay, say sh700 000 (about 180 USD), I would leaveʼ* (Female health worker, Kamuli district).

*ʽ* *I planned to have my private clinic when I started to work, which I have achieved. It is the key reason why I am still here. It gives me some side income to pay school fees for my family and also enjoy lifeʼ* (Male health worker, Kamuli district).

## Discussion

The factors influencing long stay in rural areas were revealed to be complex, often manifesting as striking a balance between the cost of leaving and the cost of living. Our discussion highlights the organizational, personal, and work-related factors, which influence long stay and the implications they may have for health workforce management.

### Organizational factors

Organizational factors which included physical features, structures and policies, personal factors, work task factors, and work climate contributed minimally to health workers’ willingness to stay, mostly by constraining health workers from moving away because of restrictive policies. Some studies have shown that factors related to hospital infrastructure and work environments enhance health worker retention [18,19,28] because not many people relish working in an environment where the infrastructure is in a critical condition or lacking [21]. Interestingly, the health workers who stayed in their posts tended to overlook poor hospital infrastructure and accept their work environment with an attitude of resignation that they could not change much. Moreover, these health workers believed that working in a difficult environment proved their mettle. This finding could be compounded by the fact that these health workers rightly perceive moving to another district to be more futile, due to the fact that infrastructural challenges are acutely experienced in most of the districts.

The availability of accommodation facilities for staff is a known motivator for health worker retention [20]. According to Wurie et al. [5], the lack of accommodation is a major deterrent to health worker retention and staff recommend that decent accommodation should be provided in order to retain staff in rural postings. Indeed, staff accommodation ensured safety of staff, reduced travel expenses and living costs and kept families together in this study, as is highlighted in other studies [5,29]. On the other hand, amidst health systems challenges of high workload and low staffing levels, staff accommodation was also viewed as a source of stress to health workers, because staying near the facility put them at risk of being required to be on duty all the time. As a result, they preferred to live far away from the hospitals. In India, a study revealed that some doctors preferred to live far away from their post location [30]. This implies that even favorably perceived health workforce incentives such as accommodation need to be monitored for their unintended effects in order to mitigate any adverse outcomes.

Heavy workload often stimulates health workers to look for better working conditions [23,31]. In neighboring Kenya, staff shortages and overwhelming workloads make health workers consider leaving their jobs [32]. The health workers in this study reported frustration with escalating workload, but it was not enough to make them quit their jobs. The health workers reported that being able to prescribe out of stock drugs provided solace, but the lack of equipment was unbearable. The lack of equipment can indeed be a limiting factor for health professionals to accept and retain positions in rural areas due to the fact that they cannot utilize their knowledge to the fullest without equipment [23]. Other studies [33,34] also report that improvements in frequency and quality of supervision, mentorship, availability of supplies, and teamwork are a consolation for health workers to be able to meet their responsibilities. According to the World Health Organization [35], good and safe working environments which possess appropriate equipment and supplies, and provide support supervision increase the recruitment and retention of health workers in rural and remote areas.

Promotion, if not done as a mere ritual, is an incentive to work in remote and rural areas [23]. Indeed promotions boosted health workers’ morale, although they were infrequent due to the paucity of long-term training opportunities and restricted wage bill. Authorities respond to the restricted wage bill by opting to recruit more junior staff to close the staffing gaps rather than promote existing staff. Subsequently, infrequent and delayed promotions usually demotivate staff [36]. There needs to be a rational and predictable promotion mechanism so as to retain health workers in rural posts [29].

Health workers emphasized the added value of being able to hold leadership positions, which not only put them in position to obtain additional revenue, but gave them access to the people in the higher echelons of government locally and nationally. Having access to additional revenue has been found to be an effective strategy in improving health worker retention [37] although financial incentives by themselves have been found to be inadequate [38]. Males had more promotion, leadership, and training opportunities than females. According to Buzuzi et al. [39]., males tend to be ‘impatient’ with the system and opt for self-funded training courses, while most females await their turn to take up training opportunities. Unfortunately, women are often unable to take the opportunities even when they arise, due to gendered family responsibilities. Subsequently, males are more able to access longer term training because they can leave their families to their spouses [40]. Promotions and leadership roles are tied to educational advancement [41]. Although females constitute a major proportion of health-care providers, they are poorly represented in management positions and at senior levels [42]. Buzuzi et al. [39] posits that barriers to training access and career development are shaped by gender roles and norms at the household and institutional level. In Uganda the possession of a professional certificate is needed for promotion, however, training opportunities in the health sector have not incorporated gender mainstreaming so as to boost female access to long-term training opportunities. Gender influences on career choices and progression are complex and require further exploration because of their potential role in influencing health-worker retention. Government health-worker training policies should make it possible for females to attain long-term training without necessarily departing from their families.

### Personal factors

The way personal factors impact health professionals’ choices could differ depending on the individual’s age and stage of career [43]. These factors are difficult to influence as strategies to retain health workers in rural areas because they fluctuate. Moreover, personal factors were the most cited reasons for long stay in this study.

Some health workers have been reported to stay in their posts because of the opportunity to be able to assist mankind [36], In our study, some health workers viewed their work as a vocation to the community, rather than just work which they are required to do [3,44]. More females than males tended to mention the ‘call to serve’ as a factor that motivated their long stay. Again a further study on the influence of gender on employment choices and motivations, as well as the social context may be required to elucidate this finding [20].

The importance of family and community ties on the retention of health workers in rural areas was highlighted in this study. Most health workers cited the need to be near family, maintain community ties, and have opportunity to invest and amass assets as reasons for their long stay in rural areas. Previous research also shows that health workers’ ‘embeddedness’ at a location is influenced by community ties, having families in a location, and opportunities to invest [44–46]. In such circumstances, the health workers choose the community first, then they choose the job indirectly [44]. Officials who recruit health workers should consider health worker preferences and attachment to communities since this may enhance their retention.

A job is liked if it is stable. Most health workers in rural areas stay in their jobs because of job security and pensions [44]. Indeed, all health workers unanimously indicated a preference for working within the public sector whether it was urban or rural mostly because of its permanent job positions. The lack of job security within the private sector has long been a deterrent for health workers in Uganda [26], and for the health workers interviewed, the stability of tenure and pension benefits clearly deterred health workers from taking up more lucrative offers. Recruitment policies should be geared towards making health workers feel more secure in their workplaces so as to inspire job commitment.

### Work stresses and coping mechanisms

The many sources of stress that make health workers demotivated or consider leaving their jobs are well documented. In this study, health workers faced work-related stress and stress related to delayed salaries. Similar to previous studies [44,47] health workers coped with the two main sources of stress in several ways. These coping strategies required the health workers to supplement their income or to be distracted.

Family, teamwork, the unction to serve, and not taking up hospital accommodation were mechanisms of coping with work-related stress while engaging in dual practice, supplementing income with project work were mechanisms of coping with low and or delayed salaries.

### Methodological consideration

We defined long-term retention as 10 years in the district. In so doing, the study might have inadvertently excluded an important cadre of health professionals – the doctors who are infrequently retained in the rural setting beyond 2–3 years. Although we have no reason to believe that the factors for long-term retention of doctors differ from those who participated in this study, we are aware that the market forces attracting doctors from the rural settings are stronger than those of our participants. Furthermore, we defined retention only in terms of time (10 years) without specifying whether this retention was voluntary or not. This could have affected our interpretation of reasons for staying because we assumed all retention was voluntary.

### Implications of the study

In general this study highlights the importance of considering both personal and organizational aspects with regard to recruitment policies aiming for long-term retention. It might be crucial for recruiters to note that personal factors might trump all other factors when it comes to long-term retention, because organizational factors such as decentralization policies and promotions are applied uniformly, regardless of where one might be within the public sector. The ability of health workers to cope in challenging environments enables their retention in these settings.

## Conclusion

Family and community ties are major factors in long-term retention and should be considered during recruitment. Barring increases in salaries, opportunities should be provided for health workers to invest in order to cope with inadequate remuneration in rural places, and to facilitate their ability to invest and bond with their communities. Job security and retirement benefits encourage health workers to stay longer at their jobs. Promotions and leadership only motivate if tied to financial benefits.

## Paper context

In Uganda, 70% of health workers serve in urban settings where 27% of the population lives. Incentives that attract and motivate health workers are documented, but incentives for long-term retention in rural settings are not adequately studied. Staffing levels in Uganda appear to be more strongly influenced by retention rather than recruitment, therefore the drivers of long-term stay remain critical.
